# Vehicular Sensor Networks: Applications, Advances and Challenges

**DOI:** 10.3390/s20133686

**Published:** 2020-07-01

**Authors:** Fatih Kurugollu, Syed Hassan Ahmed, Rasheed Hussain, Farhan Ahmad, Chaker Abdelaziz Kerrache

**Affiliations:** 1 Cyber Security Research Group, College of Engineering and Technology, University of Derby, Derby DE22 3AW, UK; f.ahmad@derby.ac.uk; 2 JMA Wireless, Liverpool, NY 13088, USA; sh.ahmed@ieee.org; 3 Institute of Information Systems, Innopolis University, 420500 Innopolis, Russia; r.hussain@innopolis.ru; 4 Department of Mathematics and Computer Science, University of Ghardaia, Ghardaia 4700, Algeria; ch.kerrache@univ-ghardaia.dz

**Keywords:** vehicular sensor networks (VSN), vehicular ad-hoc networks (VANET), security, privacy and trust, cyber security, multimedia and cellular communication, emerging IoT applications in VANET and VSN, blockchain within VANET and VSN

## Abstract

Vehicular sensor networks (VSN) provide a new paradigm for transportation technology and demonstrate massive potential to improve the transportation environment due to the unlimited power supply of the vehicles and resulting minimum energy constraints. This special issue is focused on the recent developments within the vehicular networks and vehicular sensor networks domain. The papers included in this Special Issue (SI) provide useful insights to the implementation, modelling, and integration of novel technologies, including blockchain, named data networking, and 5G, to name a few, within vehicular networks and VSN.

## 1. Introduction

Recent years have witnessed tremendous growth in connected vehicles due to the major interest in vehicular ad-hoc networks (VANET) technology from both the research and industrial communities. VANET involves the generation of data from on-board sensors and its dissemination in other vehicles via vehicle-to-everything (V2X) communication, thus resulting in numerous applications such as steep-curve warnings. However, to increase the scope of applications, VANET has to integrate various technologies including sensor networks, which results in a new paradigm, commonly known as vehicular sensor networks (VSN).

Unlike traditional sensor networks, every node (vehicle) in VSN is equipped with various sensing (distance sensors, Global Positioning System GPS, and cameras), storage, and communicating capabilities, which can provide a wide range of applications including environmental surveillance and traffic monitoring, etc. VSN has the potential to improve transportation technology and the transportation environment due to its unlimited power supply and resulting in minimum energy constraints. However, VSN faces numerous challenges in terms of its design, implementation, network scalability, reliability, and deployment over large-scale networks, which need to be addressed before it is realised.

## 2. Contributions

In this special issue, we collected and compiled twelve outstanding contributions focusing on various aspects, including its modelling, security, trust management, test-bed implementation of vehicular networks, and VSN technology. In the following, a brief summary of each accepted paper is provided to encourage the readers.

In the first paper, the authors emphasize the importance of software-defined networks (SDNs) and cellular networks in the realization of vehicular networks [1]. The paper provides an overview of the existing cellular network-based solutions for vehicle-to-everything (V2X) communication. Furthermore, the paper also discusses the existing architectures for integrating cellular networks with vehicular networks. Based on the discussed architectures, the role of SDN and its features are discussed for realizing V2X communication. Without loss of generality, the primary focus of this paper is on software-defined vehicular networks (SDVNs). The authors took different architectures and their implementations and carried out a comparative analysis of these techniques to define elements that are essential for the design of SDVNs. Overall, the paper provides the features of different implementations pertaining to SDVNs.

Hyogon and Kim [2] cover a very important topic of content trust in the safety-critical applications where a vehicle receives a safety message which is then used by a decision-support system to trigger a designated action by the vehicle which could be, for instance, deceleration, emergency brake, and so on. In this regard, it is critically important to perform a plausibility check on the content of the received message. This paper discusses the existing plausibility-based mechanisms to provide content trust in vehicular networks. The paper proposes a beacon-based ‘whispering’ approach where low-power beacon messages are used to verify the neighbors and then decide whether to trust the content of the received message or not. This work is also closely related to the Sybil attack where illusion is created by creating fake nodes. The low-power in the beacon messages is an important contribution where the authors take into account the fact that using low power in the beacon could be beneficial for proving the proximity of the neighbors. Thus, they could be used to check the plausibility of the received message contents.

Salman et al. [3] addressed the problem of data dissemination in smart cities. In smart cities, a massive amount of data is generated by a huge number of data sources and there is a need for efficient mechanisms to collect data and send it to the control units for further processing. The vehicular network is one option to carry out such tasks where vehicles are used as data carriers. Instead of using dedicated mechanisms for data sharing with the control units, in this work, the authors use mobility patterns of the vehicles and leverage them for data dissemination as well. This phenomenon not only increases efficiency, but also reduces the carbon emission because of the massive data dissemination in smart cities. This paper develops a mathematical model to measure the degree of data offloading by taking into account the communication between vehicles and RSUs. The software then develops an algorithm to select the data dissemination nodes in an energy-efficient way to offload data to the control centers in smart cities. The paper also takes Auckland city as an example to validate the efficacy of the proposed data dissemination schemes.

Likewise, Hadiwardoyo et al. [4] have targeted another very interesting idea of bringing UAV communication to the connected vehicles domain. The rationale behind this idea is to bring connectivity and positioning services among cars which are non-line of sight due to the terrain and infrastructure hazards. In this paper, the authors have modelled UAV to act as a mobile roadside unit (RSU) and proposed an algorithm to achieve good visibility levels towards the current location of a target car. The positioning technique proposed optimizes the position of the UAV, defining its best altitude so that it can avoid terrain blockages.

On the other hand, works in [5,6] studied the cache management problem in vehicular networks over the new paradigm of informationcentric networking. In particular, Amadeo et al. [5] presented the benefits of tracking the content lifetime in named data networking (NDN) packets to prevent stale information from becoming disseminated in the vehicular network. Furthermore, they also proposed an efficient NDN-compliant caching strategy that accounts for the content lifetime for both replacement purposes and caching decisions.

Unlike the conventional case when all nodes store copies of the popular data, Meng et al. [6] proposed a new distributed caching strategy at the edge of the network in vehicular social networks environments to reduce the number of overall data dissemination problems. The proposed strategy called DCS is studied comparatively against a number of conventional caching strategies and the presented results show its efficiency in terms of memory consumption, path stretch ratio, cache hit ratio, and content eviction ratio.

As the VSNs have become popular over time, a massive increase in the data traffic has been observed from the connected vehicles. This data traffic is usually transferred via 5G mobile networks. Therefore, the device-to-device (D2D) communication mechanisms have also been studied recently to make the resultant communication performance better for vehicles within 5G-based VSN. However, D2D communications are prone to network interference. The interference is usually reduced via different interference management techniques including power controls and optimal mode controls. Hyebin and Lim [7] proposed a novel technique using joint power-control and optimal mode-selection via reinforcement learning which provides energy optimization within VSN. Extensive simulations are carried out to validate the proposals, which suggests that the proposed scheme performs best in terms of achievable data rate and system energy efficiency.

Recently, blockchain has been introduced as a novel mechanism to achieve security in the vehicular networks. In particular, Lewis et al. [8] proposed a novel blockchain-based event driven message protocol dissemination framework for vehicular networks using edge computing in the 5G cellular architecture. In this proposed architecture, the authors used a lightweight multi-receiver signcryption scheme without pairing to ensure low-time consuming operations, security, privacy and access control in the network. Further, the architecture uses a private blockchain system in the network for reliability and auditability purposes. The proposed architecture is validated, and the efficiency of the protocol is evaluated in terms of overall security, communication and computational costs.

On the other hand, Chuanyi and Li [9] have explored a completely different yet timely topic of resource-limited wireless sensor networks and cluster formation. Communication protocols in WSNs are very much in numbers, however, most of those schemes failed to consider the resource efficiency issue of the trusted computing itself. In this new study, the proposed cross-validation scheme computes trust values among cluster members and cluster heads. The proposed trust management scheme is believed to be fast and resource-saving as it enables the cooperation of nodes in an efficient way. Further, the trust model is effective against collaborative attacks as well. Through extensive simulations, a proof of concept is provided to further validate the scheme.

Geetanjali et al. [10] discussed the issue of malicious intruders in the vehicular networks. The main aim of these intruders is to mislead the overall communication by disseminating malicious content to both connected and autonomous vehicles in the network. To address these issues, the authors proposed a novel blockchain-based framework which can ensure the secrecy and transparency in the network as the information is stored and traced in the backend blockchain. This framework is validated across various security criteria including fake requests of the user, compromise of smart devices, probabilistic authentication scenarios and alteration in stored user’s ratings. The proposed framework achieved the success rate of 79% over the baseline method which shows that this blockchain-based framework can be utilized to secure the connected and autonomous vehicles in the network.

Moving further, Sani et al. [11] have proposed a new MAC protocol for wireless sensor networks (WSN) that comprises a new Initial Control Frame Message, Traffic Estimation Function, Control Frame Message, and Adaptive Function. Using these four data structures, through different simulations in OMNET++, the protocol achieves higher latency and less energy consumption.

Farman et al. [12] proposed an efficient and accurate barrier control system to recognize the vehicle license plate using sensor platforms. As the license plate has various backgrounds, colors and fonts, it is extremely challenging to recognize the license plate of the vehicle accurately. In the proposed method, a vehicle is detected automatically using ultrasonic sensors and then image-based recognition is utilized with the aim to recognize a vehicle license plate. The authors implemented this mechanism on a PC running MATLAB and Raspberry Pi running Python and OpenCV. The results showed high accuracy where several license plates were used and nearly 93% of license plates were identified correctly.
